# The Tumor Microenvironment as a Therapeutic Target in Cutaneous T Cell Lymphoma

**DOI:** 10.3390/cancers16193368

**Published:** 2024-10-01

**Authors:** Louis Boafo Kwantwi, Steven T. Rosen, Christiane Querfeld

**Affiliations:** 1 Department of Pathology, City of Hope Medical Center, Duarte, CA 91010, USA; lkwantwi@neomed.edu; 2 Beckman Research Institute, Duarte, CA 91010, USA; srosen@coh.org; 3 Department of Anatomy and Neurobiology, College of Medicine, Northeast Ohio Medical University, Rootstown, OH 44272, USA; 4 Department of Hematology & Hematopoietic Cell Transplantation, City of Hope Medical Center, Duarte, CA 91010, USA; 5 Division of Dermatology, City of Hope Medical Center, Duarte, CA 91010, USA

**Keywords:** mycosis fungoides, Sézary syndrome, tumor microenvironment, cutaneous T cell lymphoma, cytokines, immune checkpoints, genetic alterations

## Abstract

**Simple Summary:**

Cutaneous T cell lymphomas (CTCLs) are a group of rare lymphoproliferative malignancies manifesting in the skin. Cutaneous T cell lymphomas are an incurable, disfiguring, and life-threatening disease. Emerging studies have implicated the surrounding cells of malignant T cells (tumor microenvironment) in the disease evolution. This has revealed that targeting the tumor microenvironment has therapeutic potential in cutaneous T cell lymphomas. This review provides a detailed insight into the contribution of the tumor microenvironment in cutaneous T cell lymphomas and the targeting strategies.

**Abstract:**

Cutaneous T cell lymphomas (CTCLs) are a heterogeneous group of non-Hodgkin lymphomas, with mycosis fungoides and Sézary syndrome being the two common subtypes. Despite the substantial improvement in early-stage diagnosis and treatments, some patients still progress to the advanced stage with an elusive underpinning mechanism. While this unsubstantiated disease mechanism coupled with diverse clinical outcomes poses challenges in disease management, emerging evidence has implicated the tumor microenvironment in the disease process, thus revealing a promising therapeutic potential of targeting the tumor microenvironment. Notably, malignant T cells can shape their microenvironment to dampen antitumor immunity, leading to Th2-dominated responses that promote tumor progression. This is largely orchestrated by alterations in cytokines expression patterns, genetic dysregulations, inhibitory effects of immune checkpoint molecules, and immunosuppressive cells. Herein, the recent insights into the determining factors in the CTCL tumor microenvironment that support their progression have been highlighted. Also, recent advances in strategies to target the CTCL tumor micromovement with the rationale of improving treatment efficacy have been discussed.

## 1. Introduction

Cutaneous T cell lymphomas are a rare form of non-Hodgkin lymphomas characterized by the accumulation of malignant CD4+ lymphocytes homing into the skin [1]. Even though the etiology is still an enigma, the highest incidence rates are found in African American and aged populations with a four-fold increase in individuals over 70 years [2,3]. Although it is a heterogenous disease, mycosis fungoides (MF) and Sézary syndrome (SS) account for 60% of all cases, making them the most studied and common subtypes [4]. MF is defined by patches, plaques, tumors, and/or erythroderma, while SS is a more aggressive and leukemic form of CTCLs characterized by erythroderma and the presence of clonally similar neoplastic T cells with cerebriform nuclei (Sézary cells) in the peripheral blood, skin, and/or lymph nodes [5,6]. The heterogeneous presentation not only makes a definitive diagnosis of CTCLs often difficult but also the selection of appropriate therapeutic options [7]. Although efforts made to understand the pathophysiology of CTCLs have led to the development of new treatment modalities for the early stage [8] and advanced stage of the disease [9,10,11], most patients develop progressive disease due to treatment failure, which makes our knowledge of the exact molecular mechanisms underpinning the disease incomplete. Given the reduced survival rates together with the increasing aggressiveness of CTCLs, studies to map the underlying mechanisms have pinpointed the critical role of the CTCL tumor microenvironment (TME) in the disease processes. Notably, the interaction between malignant T cells and their niche via cytokines dysregulations, genetic alterations, and immune cells infiltrating the microenvironment have become crucial determinants in tumor initiation, metastasis, therapeutic resistance, and other hallmarks of CTCLs [1,12,13]. Therefore, this review aims to elucidate the molecular interaction between CTCLs and their microenvironment and evaluate how such an interaction affects the fate of malignant T cells. In addition, insights into the recent advances in strategies to target the TME are highlighted.

## 2. Role of CTCL Tumor Microenvironment in CTCL Progression

The CTCL TME is composed of malignant T cells, endothelial cells, fibroblast, keratinocytes, and immune cells, including macrophages, monocytes, B cells, neutrophils, mast cells, eosinophils, natural killer cells, dendritic cells, T cells, myeloid-derived suppressor cells, and regulatory T cells [1,14,15]. The cellular communication mediated by tumor-derived factors alters the physiological role of antitumor immune cells, which shields CTCL cells from therapeutic agents, hence promoting their progression. Notably, the hostile nature of the TME, which is partly attributed to hypoxia [16], causes endothelial cells in the CTCL TME to proliferate and form new vessels, thereby promoting tumor progression [17,18]. Moreover, malignant T cells also activate stem cells and epithelial-to-mesenchymal transition to support their continuous renewal and differentiation [12]. Hence, the microenvironmental niche of CTCLs contributes to all facets of the disease processes, including immunosuppression [19,20,21], therapeutic resistance [22,23], apoptosis resistance [21,24,25,26,27], invasion and migration [28,29,30,31], angiogenesis [17,18,32], and tumor proliferation [33,34,35,36]. 

### 2.1. The Immune Tumor Microenvironment and CTCL Progression

#### 2.1.1. Macrophages

Macrophages are one of the major leukocytes infiltrating the tumor microenvironment. Depending on the prevailing conditions in the TME, macrophages can be polarized into M1 and M2 phenotypes. Functionally, tumor-associated macrophages (TAMs) designated as M1 exhibit pro-inflammatory and antitumor functions, while M2 macrophages are anti-inflammatory with tumor-promoting functions [37]. Indeed, it has been found that while the early stage of MF is characterized by the high infiltration of M1 macrophages, M2 macrophages predominate in advanced tumors [38]. In the large cell transformation of MF, miR-708 downregulation and upregulation of miR-146a and miR-21 were associated with the infiltration of M2 macrophages, suggesting their immunosuppressive role in the TME [39]. Furthermore, both CD68+ and CD163+ macrophages are associated with the advanced stage of CTCLs [40,41].

In several mechanistic studies, macrophages polarized by CTCL TME tend to support CTCL progression through the expression of cytokines and growth factors [42,43,44,45]. For example, inflammatory cytokines, including CXCL5, CCL13, and IL10, produced by periostin-induced TAMs were found to create an immunosuppressive tumor microenvironment, leading to MF development [42]. Furthermore, M2 macrophage cocultured with CTCL cells upregulated S100A9/TLR4 via NF-kB to induce apoptosis resistance, leading to CTCL progression [19]. More recently, Han et al. showed that PD-1+ M2 macrophages induced by CTCL TME through the NF-kB/STAT/JAK pathway can impair the phagocytic activity of macrophages, promoting CTCL growth [46]. Macrophages are also important drivers of angiogenesis in CTCLs, as described by Wu et al. [47]. The depletion of M2-like TAMs delayed CTCL development in xenograft mouse models, supporting their role in CTCL tumorigenesis [47].

**Figure 1:**
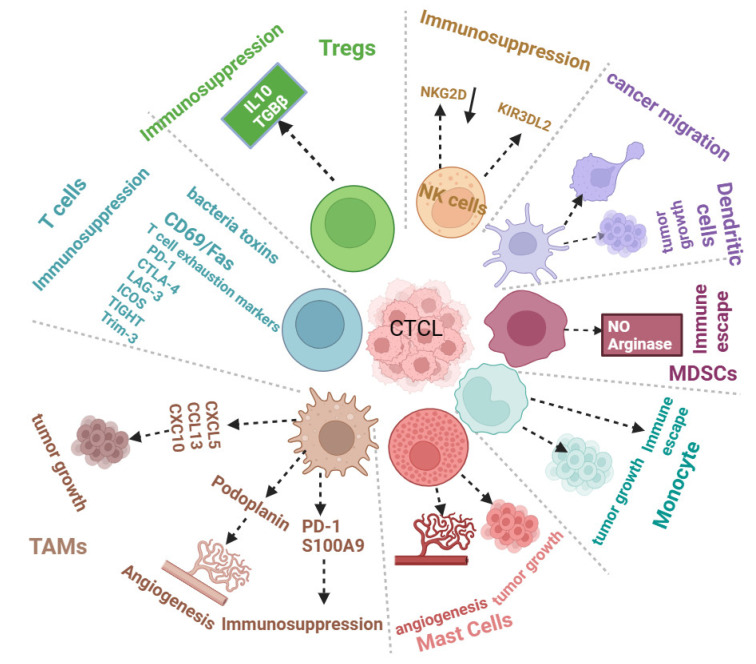
CTCL TME negatively regulates the tumor immune microenvironment to support CTCL progression. Immune cells infiltrating the CTCL tumor microenvironment promote angiogenesis, tumor growth, migration, and immunosuppression.

Regarding monocytes, evidence suggests that malignant T cells can recruit monocytes via a CCL5-dependent manner to promote the survival of CTCL cells [48]. Furthermore, monocytes can interact with malignant T cells to promote immunosuppression and CTCL progression [19], as shown in Figure 1.

#### 2.1.2. Mast Cells

Mast cells infiltrating the CTCL TME have been established as key players in the disease processes. In CTCL lesions, increased mast cells not only show a positive relationship with tumor stage but also microvessel density, suggesting their role in inducing angiogenesis. In support of this, delayed tumor growth was found in a cutaneous lymphoma mouse model deficient in mast cells [49]. In MF, a high number of mast cells and tryptase are drivers of itch and MF disease severity [50]. In contrast, Eder et al. found higher mast cells in clinical stage IA and IB patients than in the IIA and IIB stages [51], suggesting that a higher number of mast cells may not necessarily reflect the stage of CTCLs.

#### 2.1.3. Eosinophils and Neutrophils

Eosinophils and neutrophils are important components of innate cells with key roles in host defense mechanisms. However, signals within the TME can alter their physiology to support tumor progression [52,53,54,55]. Studies have shown that a high density of eosinophils either in blood [56] or skin lesions is associated with the aggressiveness of the disease [14,56]. Similar to other innate immune cells, the activation of eosinophils drives inflammation in CTCLs to accelerate disease progression [14,57]. In a study aimed at elucidating eosinophils-activating factors in CTCL TME, IL5 and high mobility BOX-1 protein (HMGB1) expressed by malignant T cells were identified as key activators of eosinophils in MF [58]. Moreover, a high infiltration of neutrophils mediated by IL17 and IL8 is linked with MF and SS disease progression [59,60].

#### 2.1.4. Dendritic Cells and Natural Killer Cells

Dendritic cells (DCs) are the most efficient antigen-presenting cells noted for capturing and presenting antigens to naïve T cells [61]. Dendritic cells can exhibit both anti-tumor and protumor functions depending on the prevailing conditions within the TME [61]. Whereas mature dendritic cells play antitumor functions, immature dendritic cells foster immune tolerance, thus promoting tumor progression [62]. Berger et al. cocultured immature dendritic cells with CTCL cells from SS patients and found that immature dendritic cells can sustain the growth of CTCLs [63]. Furthermore, DCs can promote the migration of SS and MF cells [64]. OX40 is a costimulatory signal that promotes T cell expansion and survival. In a recent study, the activation of benign T cells by OX40L+CD40L+ dendritic cells stimulated inflammation and the release of tumorigenic signals to CTCLs [65]. Natural killer cells (NKs) are lymphoid members of the innate immune system playing cytotoxic functions similar to CD8+ T cells. In CTCLs, malignant T cells from SS patients can reduce CD16+CD56^dim^ NK cells and downregulate NKG2D, the main activator of antitumor activity in NK cells, to promote their escape from NK-induced antitumor immunity [66]. Additionally, the increased expression of NK cell receptor KIR3DL2 has been found in MF [67].

#### 2.1.5. Myeloid-Derived Suppressor Cells (MDSCs)

MDSCs represent a heterogeneous population of immune cells implicated in many pathologic conditions, including cancers [15,68]. In CTCLs, MDSC accumulation is linked with worse clinical outcomes in patients [69] and the advanced stage of SS and MF [70]. Furthermore, MDSCs have been shown to express high levels of arginase and nitric oxide (NO) to potentiate immunosuppression and CTCL progression [20]. Maliniemi et al. showed that CD33+ myeloid suppressor cells express indoleamine 2,3-deoxygenase 1, an immune checkpoint molecule, supporting their role in immunosuppression [71].

#### 2.1.6. Tumor-Infiltrating Lymphocytes

Tumor-infiltrating lymphocytes are composed of heterogeneous immune cells with the primary function of clearing tumor cells. However, immunosuppressive factors in the TME hampers their function to facilitate tumor escape and progression [72]. Largely, markers associated with T cell exhaustion, including PD-1, CTLA-4, LAG-3, TIGIT, and TIM-3 [73,74], have been shown as crucial players in cancer-mediated immunosuppression in CTCLs. Furthermore, emerging evidence shows that microbiota present in CTCL TME can negatively regulate antitumor immunity, as demonstrated by Blümel et al. [75]. Here, authors established that staphylococcal alpha-toxin can promote the escape of CTCL cells from CD8+ T cell-mediated killing, hence facilitating SS progression [75]. Additionally, the cytotoxic functions of lymphocytes can be impaired through CTCL-mediated inhibition of cytokines involved in T cell priming. For example, SS cells can attenuate the cytotoxic functions of CD8+ T cells by suppressing their responsiveness to IL10 [76]. Furthermore, Zhen et al. have established that increased expression of miR-155, -130, and -21 in Hut78 and Myla cell lines can induce CD8+ T cell exhaustion, leading to CTCL progression [77].

B cells infiltrating the CTCL tumor microenvironment contribute to the pathophysiology of the disease. It has been shown that there is a high infiltration of B cells in MF patients compared to healthy controls [78]. Additionally, the high infiltration of B cells correlates positively with MF progression [78]. Functionally, B cells in the MF tumor microenvironment release immunosuppressive cytokines, contributing significantly to tumor cell growth, dissemination, angiogenesis, and immunosuppression [13].

#### 2.1.7. Regulatory T Cells

In MF, Tregs have diverse functions with contrasting roles having been reported. While the early patch stage of MF is associated with high Treg numbers, a low number of FOXP3+ cells are found in the advanced stage [16,79,80,81]. Indeed, the reduced expression of FOXP3+ cells relative to CD3+ T cells in the early stages of MF correlates with the disease progression [82]. On the contrary, a high infiltration of Tregs correlates with good clinical outcomes in MF patients [82]. In SS, IL10 and TGF-β secreted by Tregs can suppress the secretion of IL2 and IFN-γ and maintain DC immaturity, leading to CTCL proliferation [83]. Similarly, high Tregs(CD4+ CD25+) in SS patients have been found to suppress the proliferation of autologous CD4+ CD25- responder T cells [20].

Evidence has shown that the microbiota in CTCL TME can augment the tumor-promoting functions of Tregs [84]. According to Willerslev-Olsen et al. Staphylococcus aureus enterotoxins (SEA) can induce FOXP3 expression in malignant SS cells via the STAT5 pathway [84].

### 2.2. Role of Cancer-Associated Fibroblasts in CTCL

**Figure 2:**
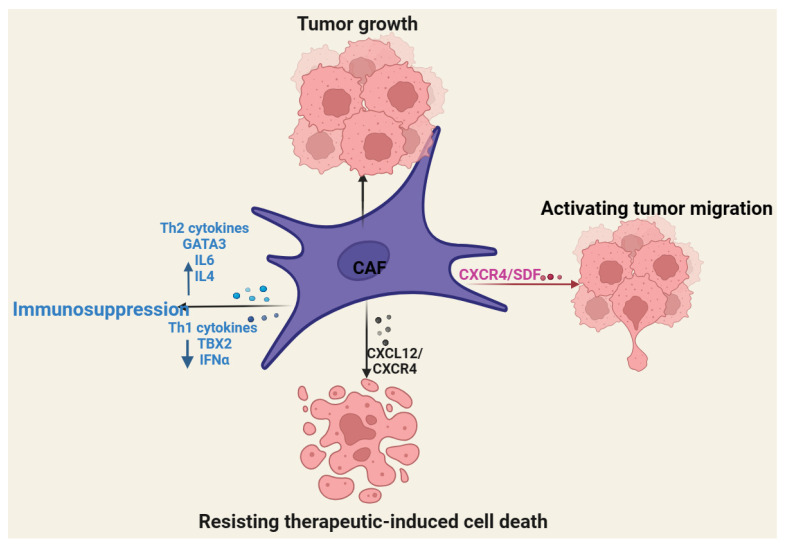
Role of cancer-associated fibroblasts in CTCLs. Cancer-associated fibroblasts promote CTCL migration, growth, apoptosis resistance, and immunosuppression.

Cancer-associated fibroblasts (CAF) are major components of the TME known to potentiate the immune escape of tumor cells [85,86]. In CTCLs, CAF can modulate the expression of biomarkers associated with CTCL pathogenesis, attenuate Th1-related cytokines, and promote the Th2-dominant microenvironment, leading to CTCL progression [87,88]. For example, MF cells were found to induce normal fibroblast to express high levels of TWIST1 and TOX and Th2 markers, such as GATA3, IL6, and IL4 but low levels of Th1 markers, IFNG and TBX2 [87] (Figure 2).

Data have shown that chemokines secreted by CAF can potentiate their tumor-promoting functions [22,29,89]. Specifically, CAF-induced CXCR4/SDF promoted SS cell migration by downregulating CD26/dipeptidyl peptidase IV [29]. Relatedly, eotaxins derived from dermal fibroblast can interact with CCR3+ lymphocytes to promote CTCL development [89]. Furthermore, CXCL12/CXCR4 secreted by MF-derived CAF was found to protect malignant T cells from doxorubicin-induced apoptosis, thereby enhancing the migration of MF cells [22]. Beksac et al. also cocultured fibroblast and malignant MF cells isolated from the skin of early-stage CTCLs and found that fibroblast can enhance the proliferation of MF cells [33].

### 2.3. Role of Vascular or Endothelial Cells in CTCLs

Angiogenesis, characterized by vascular or lymphatic vessel formation, is an important process for tumor dissemination [90,91,92]. Several molecular and correlative studies have detailed the indispensable role of endothelial cells and their related markers in CTCL pathogenesis. In MF and SS, the expression levels of VEGFR-3, VEGF-C, and other angiogenic markers, such as CD31, podoplanin, and LYVE-1, correlate significantly with disease progression [93,94,95,96]. Besides tissue expressions, serum levels of VEGF-A reflect the severity of itching in MF and SS patients [97]. Furthermore, a positive correlation between podoplanin expression and lymphatic vessel density in malignant T cells has been linked to tumor aggressiveness and advanced stage of MF [98]. Moreover, intertumoral SOX18, a marker of neovascularization, correlates with MF disease progression, cutaneous involvement, and metastasis [28].

**Figure 3:**
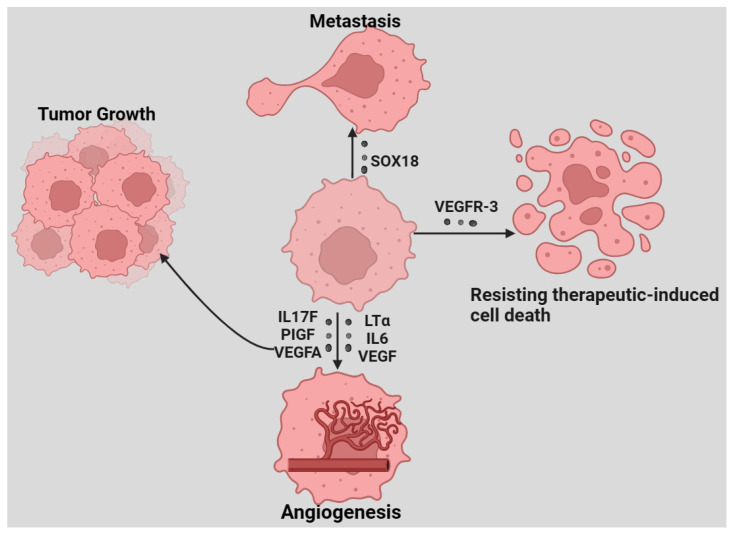
Contribution of endothelial cells to CTCLs. Endothelial cells promote angiogenesis, tumor metastasis, growth, and apoptosis resistance.

Mechanistically, in situ expression of LTα driven by the aberrant activation of the JAK3/STAT5 pathway acts in an autocrine fashion via TNF-alpha receptor 2 to induce IL6 expression in malignant T cells, which together with VEGF induce tube formation and endothelial cell sprouting [18]. According to Lauenborg et al. IL17F derived from Myla supernatant can stimulate angiogenesis through tube formation and sprouting to facilitate CTCL progression [17]. Furthermore, placental growth factor (PlGF) and VEGF-A expressed in CTCL skin were found to promote tumor growth via tumor vasculature formation [32]. The same study found serum levels of PIG4 to correlate with MF/SS disease severity, suggesting a possible utility of PIG4 either as a biomarker or potential therapeutic target [32]. Additionally, VEGFR-3 expressed in CTCL cell lines and a xenograft mouse model of MF exhibited a protective effect towards the suberoylanilide hydroxamic acid (SAHA)-mediated inhibition of tumor cells, hence promoting tumor progression [23], as shown in Figure 3.

## 3. Molecular Mechanisms of CTCL Immune Evasion

**Figure 4:**
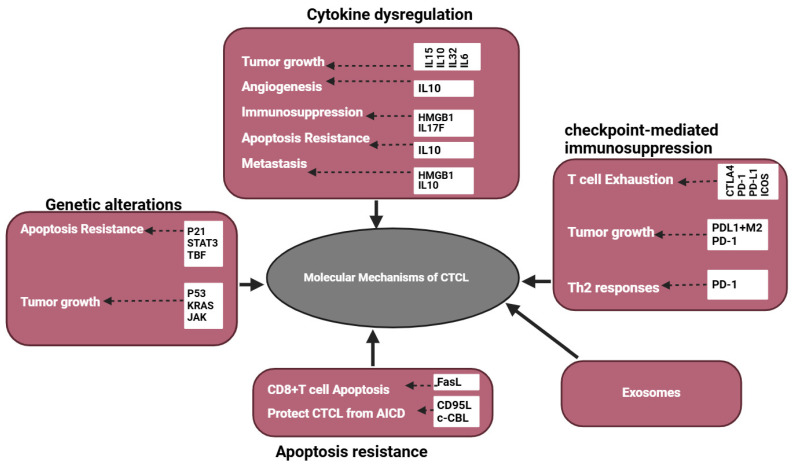
Molecular mechanisms of CTCL progression: CTCL TME influences the malignant transformation of CTCLs through genetic alterations, apoptosis resistance, immune checkpoint-mediated immunosuppression, cytokine dysregulations, and exosome secretions.

Immune evasion of CTCLs involves several mechanisms, including the secretion of immunosuppressive factors, such as cytokines and exosomal cargos, genetic alterations, immune checkpoint-mediated T cell inhibition, and apoptosis resistance, as shown in Figure 4.

### 3.1. Apoptosis Resistance

Apoptosis evasion is a hallmark of cancer progression. This process is characterized by a decrease in the function of pro-apoptotic proteins and/or an increase in anti-apoptotic proteins. They can block cell death signals, hence promoting apoptosis resistance [99]. Fas ligand (FasL) expressed on cytotoxic T cells plays an important role in the Fas-mediated killing of tumor cells. However, FasL expressed on tumor cells can counterattack the tumor-killing abilities of tumor-infiltrating lymphocytes [100]. In support of this, Ni et al. have found that FasL expressed by malignant T cells and epidermal keratinocytes can induce the apoptosis of CD8+ T cells and MF progression [101]. Indeed, fewer CD8+ T cells appear to be distributed in the vicinity of FasL-positive tumor cells.

CTCL progression is dependent on their ability to escape activation-induced cell death (AICD) [24,25,26,27]. According to Klemke et al., malignant T cells from SS patients show reduced surface expression of CD95L, an apoptosis-inducing ligand, upon TCR stimulation, leading to AICD resistance [24]. Relatedly, the overexpression of E3 ubiquitin ligase c-CBL in CTCL cells inhibits AICD [25]. Genomic instability [102], mutations in genes [103], dysregulation of cytokines, and signaling pathways [104,105] are other mechanisms implicated in apoptosis resistance in CTCLs.

### 3.2. Cytokine Dysregulation in CTCLs

The pathophysiology of cancers is impacted considerably by the cytokine milieu of its environment [91,106,107,108]. Specifically, alterations in cytokines such as IL32, IL22, IL17F, IL17A, IL16, IL15, and HMGB1 [109,110] can create an immunosuppressive tumor microenvironment in CTCLs to facilitate immune evasion and tumor progression. According to Ito et al., C-C motif chemokine ligand 20 (CCL20) induced by IL22/IL22RA1 axis interacts with CCR6 receptor to promote migration and distant organ metastasis of CTCLs [31]. Intriguingly, increased CCL20 in clinical samples correlates positively with CTCL progression [111]. The elevated expression of 1L10 in malignant T cells facilitates tumor growth in vivo through IL10-mediated macrophage infiltration and M2 polarization [112]. Moreover, IL10 expressed by malignant T cells can impair the differentiation of monocytes to matured DCs, leading to antitumor suppression [113]. In a similar study, IKZF2-induced IL10 expression in malignant T cells was found to dampen the antitumor immunity of MHC II molecules, hence promoting apoptosis resistance and CTCL progression [21]. Indeed, increased 1L10 expression is associated with the clinical course of MF [21,112]. Ohmatsu et al. have indicated that the increased mRNA expression of IL32 not only predicts MF severity but can enhance the proliferation of CTCL cells [114]. Additionally, IL32 is known to upregulate survival genes [114,115], important for the initiation, maintenance, and progression of CTCLs [116]. IL16 and thymic stromal lymphopoietin (TSLP) expressed in the early stages of MF not only enhances the infiltration of malignant T cells into the skin but also contributes to CTCL proliferation [30]. The contribution of IL31 to CTCL pathogenesis appears to be diverse. While elevated serum levels of IL31 correlate positively with advanced disease stage [60] and pruritus [117], Santen et al. found low levels of IL31 in pruritic folliculotropic (FMF) but no expression in non-pruritic patients (MF) [118]. According to Mishra et al., the overexpression of IL15 by CD4+ T cells is associated with histone deacetylase histone (HDAC)1/6 upregulation and miRNA-21 activation, promoting CTCL progression [119]. Furthermore, the activation of mTORC1 by IL15 and IL2 in malignant CD4+ T cells promoted CTCL proliferation [120]. In addition to the above, Thode et al. have demonstrated that IL15 expressed by malignant T cells activates epidermal keratinocytes to promote CTCL proliferation [121]. It is interesting to note that while IL15 expression in skin-homing CD4+ T cells and peripheral blood CD4+ T cells correlated with CTCL disease progression [119], no correlation was found between IL15 miRNA expression in malignant T cells and CTCL advancement [122]. Additionally, IL17F induced by malignant T cells has been shown to promote the malignant transformation of MF cells and angiogenesis in CTCLs [17]. Senda and coworkers assessed the role of HMGB1 in CTCLs and found that high levels of HMGB1 in skin lesions and sera are associated with increased Th2 immune response and the induction of angiogenesis [123].

### 3.3. Genetic Alterations in CTCLs

Genetic alterations, including somatic mutation and mutagenic pathways, are important regulators of several cancer types, including CTCLs [124]. Evidence indicates that p53 mutation is linked with MF progression and predicts poor survival in patients [34,125]. Consequentially, p53 mutation status has been proposed as a possible biomarker to stratify patients at risk of advanced MF disease [125]. In a more mechanistic study, the dysregulation of p53 function has been shown to protect CTCL cells from apoptosis [126,127]. Several lines of evidence have shown that p21 dysregulation is associated with increased proliferation of CTCL cells [35,36,128,129]. Moreover, KRAS mutation promotes apoptosis resistance and predicts poor prognosis in MF patients [130,131]. In MF and SS, disease progression can be potentiated by alterations in CARD11 [103], TNFRSF1B [132], PLCG1 [133], and KIT [134]. Additionally, evidence from several whole-genomic and whole-exon sequencing studies suggests that mutation in NOTCH2 [34], TNFRSF1B, CTLA4-CD28 fusion [132], RB1, PTEN, DNMT3A, CDKN1B [103], CARD11, CDKN2A, and CCR4 [135] can promote CTCL progression. Although mutations in these genes are not frequently encountered in MF and SS patients, they can serve as potential therapeutic targets for CTCL patients. According to McGirt et al., JAK3 mutation in MF cells can induce apoptosis resistance and enhance CTCL proliferation [34].

Cancer-associated microbiota are important players in cancer progression [107,136,137]. In a study by Willerslev-Olsen et al., staphylococcal enterotoxin A (SEA) cocultured with non-malignant T cells was found to activate the STAT3/JAK3 pathway and induce IL17 expression [137]. Relatedly, IL17 and IL22 induced by STAT3 hyperactivation in a bacterial-dominated environment were found to enhance the proliferation of CTCL cells [136]. In a genomic analysis conducted in mice and humans, genetic instability mediated by a mutation in telomere-binding factor (TBF) was linked to CTCL development [138]. 

### 3.4. Immune Checkpoint-Mediated Suppression of T Cells

Increased expression of immune checkpoint molecules, including CTLA-4 [73], PD-1 [21,139,140], PD-L1 [73,139,141], and ICOS [73,141], on malignant T cells correlates positively with the advanced disease stage of CTCLs. Detailed insight has revealed that PD-L1 expression in CTCL cell lines can induce M2 macrophages to promote CTCL growth [142]. Furthermore, it has been found that increased PD-1 can impair antitumor immune response and promote Th2 responses, which facilitate CTCL tumor growth [143,144]. The available evidence supports the notion that reversing T cell exhaustion is key to restoring T cell function. Although this has largely been welcomed as a potential therapeutic strategy, an integrated genomic analysis in humans and a mice model of T cell lymphomas has found that, while the loss of PD-1 function promotes the reversal of T cell exhaustion, this is associated with FOXM1-mediated transcriptional signature, leading to poor prognostic outcomes in SS and MF patients [145].

### 3.5. Exosomes in CTCLs

Studies on exosomes have demonstrated their involvement in all aspects of tumorigenesis, including invasion and migration, angiogenesis induction, and tumor escape from immunosurveillance [146,147,148]. In the CTCL context, the available evidence indicates that miR- 155 derived from MF cell lines can enhance the migratory effect of MF cells. Interestingly, plasma exosomes from MF patients enhances the migration of normal peripheral blood mononuclear cells in a coculture system [149]. However, considering the large body of evidence on the diverse role of exosomes in tumorigenesis across several cancer types, further insights are required to fully understand the role of exosomes in CTCLs.

## 4. Advances in Strategies to Target CTCLs

The past few years have seen a great improvement in cancer treatment through a combination of agents or drugs targeting the CTCL tumor microenvironment. Specifically, targeting immune evasion mechanisms of malignant T cells and other populations within the TME contributing to CTCL tumorigenesis is promising. Immune checkpoint inhibitors, including pembrolizumab, durvalumab, and ontorpacept (TTI-621; SIRPα-IgG1 Fc), have shown significant antitumor activity with durable and long-lasting responses with manageable toxicity profiles in CTCL patients [150,151,152,153,154]. The intralesional application of ontorpacept (TTI-621) has led to activity in adjacent or distal non-injected lesions, suggesting systemic and locoregional abscopal effects [153]. Studies have shown that anti-PD-L1 (durvalumab), lenalidomide, and TTI-621 can re-program M2 macrophages to boost antitumor functions against CTCL cells [46,142]. Furthermore, the depletion of macrophages in a CTCL murine model using CCR2 inhibitors can synergize with anti-PD-1 to suppress tumor growth [155]. Even in some refractory SS patients, anti-PD-1 in combination with HDAC inhibitors can promote durable clinical response [156]. In a Phase1/2 trial of anti-PD-L1 (durvalumab) and lenalidomide in CTCL patients, Querfeld et al. showed that durvalumab and lenalidomide are associated with significant clinical activity in refractory and advanced patients [157].

Given the role of cytokines and chemokines in CTCL pathogenesis, studies exploring their therapeutic potential on the CTCL TME have also emerged [151,158]. EQ101 (formerly known as BNZ-1) is a synthetic peptide, designed to selectively inhibit IL-2, IL-9, and IL-15 binding to the common gamma chain (γc) signaling receptor, leading to the depletion of Tregs and tumor growth suppression [159]. Moreover, denileukin diftitox, a recombinant fusion protein of IL2 and diphtheria toxin, targets the IL2 receptor on malignant T cells and Tregs [20]. The reengineered drug denileukin difitox-cxdl (E7777) shows improved safety and tolerability and was FDA-approved in August 2024 for relapsed/refractory CTCLs. Mogamulizumab, which is a humanized anti-CCR4, exhibits potent clinical efficacy against CCR4-positive CTCLs and other T cell lymphomas and was shown to efficiently decrease Tregs, leading to CTCL growth inhibition [160]. KIR3DL2 expression is upregulated on all subtypes of CTCLs. Anti-KIR3DL2 monoclonal antibody (IPH4102) has shown promise in depleting the KIR3DL2 receptor in malignant T cells of CTCL patients [161]. IPH4102 has been shown to recruit human effector NK cells as well as macrophages to eliminate KIR3DL2^+^ T cells via antibody-dependent cell cytotoxicity and antibody-dependent cell phagocytosis, respectively. Wang et al. compared the efficacy of CCR4-IL2 bispecific immunotoxin with brentuximab. Using an immunodeficient NSG mouse model of CTCLs, the study found that CCR4-IL2 bispecific immunotoxin was more effective in prolonging survival than brentuximab [158].

## 5. Conclusions

Malignant T cells can turn their environment into a hospitable home to promote their survival, growth, and progression. Hence, in our quest to uncover the therapeutic potential of CTCL TME, agents or drugs should not only target malignant T cells but interfere with their key defensive mechanisms and abrogate their ability to evade the antitumor immune response. To this end, the reprogramming of protumorigenic immune cells to gain their antitumor functions and apoptosis induction of CTLC cells holds promise in CTCL treatment. Such a holistic approach will open new opportunities in the treatment of relapsing and refractory CTCL patients to yield durable clinical responses.
